# The Distribution and Identity of Edaphic Fungi in the McMurdo Dry Valleys

**DOI:** 10.3390/biology3030466

**Published:** 2014-07-30

**Authors:** Lisa L. Dreesens, Charles K. Lee, S. Craig Cary

**Affiliations:** 1 International Centre for Terrestrial Antarctic Research, University of Waikato, Hamilton 3216, New Zealand; E-Mails: lisa.laura1991@hotmail.com (L.L.D.); cklee@waikato.ac.nz (C.K.L.); 2 College of Earth, Ocean and Environment, University of Delaware, Lewes, DE 19958, USA

**Keywords:** Antarctica, fungi, Dry Valleys, soil, biogeography, microbial ecology

## Abstract

Contrary to earlier assumptions, molecular evidence has demonstrated the presence of diverse and localized soil bacterial communities in the McMurdo Dry Valleys of Antarctica. Meanwhile, it remains unclear whether fungal signals so far detected in Dry Valley soils using both culture-based and molecular techniques represent adapted and ecologically active biomass or spores transported by wind. Through a systematic and quantitative molecular survey, we identified significant heterogeneities in soil fungal communities across the Dry Valleys that robustly correlate with heterogeneities in soil physicochemical properties. Community fingerprinting analysis and 454 pyrosequencing of the fungal ribosomal intergenic spacer region revealed different levels of heterogeneity in fungal diversity within individual Dry Valleys and a surprising abundance of Chytridiomycota species, whereas previous studies suggested that Dry Valley soils were dominated by Ascomycota and Basidiomycota. Critically, we identified significant differences in fungal community composition and structure of adjacent sites with no obvious barrier to aeolian transport between them. These findings suggest that edaphic fungi of the Antarctic Dry Valleys are adapted to local environments and represent an ecologically relevant (and possibly important) heterotrophic component of the ecosystem.

## 1. Introduction

Located between the Polar Plateau and Ross Sea in Southern Victoria Land, the McMurdo Dry Valleys (hereinafter the Dry Valleys) are the largest contiguous ice-free area on the Antarctic continent. Dry Valley soils are known as some of the oldest, coldest, driest, and most oligotrophic soils on Earth [1]; consequently, the Dry Valley ecosystem is characterized by a lack of nutrients [2], low precipitation levels and biologically available water [3,4,5], high levels of salinity [6,7,8], large temperature fluctuations [5,9,10], steep chemical and biological gradients [11], and high incidence of UV-solar radiation [12,13,14]. Early studies suggested that Dry Valley soils contained very little microbial biota [1], but recent molecular evidence has demonstrated the presence of diverse and heterogeneous bacterial communities potentially driven by steep physicochemical gradients [1,10,15,16,17,18,19]. In contrast, comparatively limited molecular evidence exists on the distribution and drivers of fungal communities in Dry Valley soils [20,21,22,23].

Fungal identification in Dry Valley soils by means of a combination of culturing and molecular tools (*i.e.*, denaturing gradient gel electrophoresis and DNA sequencing) has detected primarily members of Dikarya (*i.e.*, Ascomycota and Basidiomycota), including both filamentous and non-filamentous species [24,25,26,27]. A survey of Dry Valley sites including Mt Flemming, Allan Hills, New Harbor, and Ross Island revealed the dominant free-living fungal genera in Dry Valley soils as *Cadophora* (Ascomycota), *Cryptococcus* (Basidiomycota), *Geomyces* (Ascomycota), and *Cladosporium* (Ascomycota) [22]. A study of cultivable fungi in Taylor Valley showed that filamentous fungi appeared to be associated with high soil pH and moisture, whereas yeasts and yeast-like fungi had wider distribution across habitats examined [23]. Basidiomycetous *Cryptocococcus* and *Leucosporidium* species were the most frequently isolated genera in a regional survey of yeasts and yeast-like fungi in the Dry Valleys [20]. The diversity of yeasts and yeast-like fungi was positively correlated with soil pH and negatively with conductivity [20]. The same study also revealed apparent segregation of *Cryptococcus* clades found in Taylor Valley and the Labyrinths of Wright Valley [20], hinting at the presence of localized communities adapted to environmental conditions, as has been reported for soil bacteria in the Dry Valleys [15]. A culture-based study of soils taken from McKelvey Valley detected no fungal colony-forming units (CFUs) in most of the samples [21], and a molecular survey of McKelvey Valley also detected no fungal signals in the soils [18]. However, sequences affiliated with genera *Dothideomycetes* (Ascomycota), *Sordariomycetes* (Ascomycota), and *Cystobasidiomycetes* (Basidiomycota) were found in endolithic and chasmolithic communities in McKelvey Valley [18]. The evidence so far suggests that the cultivable components of Dry Valley fungal communities are dominated by ascomycetous and basidiomycetous species, although their biogeography and factors that shape their distribution in the Dry Valleys remain unclear due to the lack of systematic and culture-independent evidence. Furthermore, the ecological relevance of fungi in Dry Valley soils remains unknown since neither cultivation nor molecular techniques can effectively distinguish active fungal cells from dormant spores.

For this study, we carried out a molecular survey of Dry Valley soil fungi at six study sites (Battleship Promontory, Upper Wright Valley, Beacon Valley, Miers Valley, Alatna Valley, and University Valley) using terminal restriction fragment length polymorphism (tRFLP) and 454 pyrosequencing analyses of the fungal ribosomal intergenic spacer. Soil physicochemical properties were also characterized to examine potential environmental drivers of fungal diversity.

## 2. Experimental

### 2.1. Sample Collection

**Figure 1:**
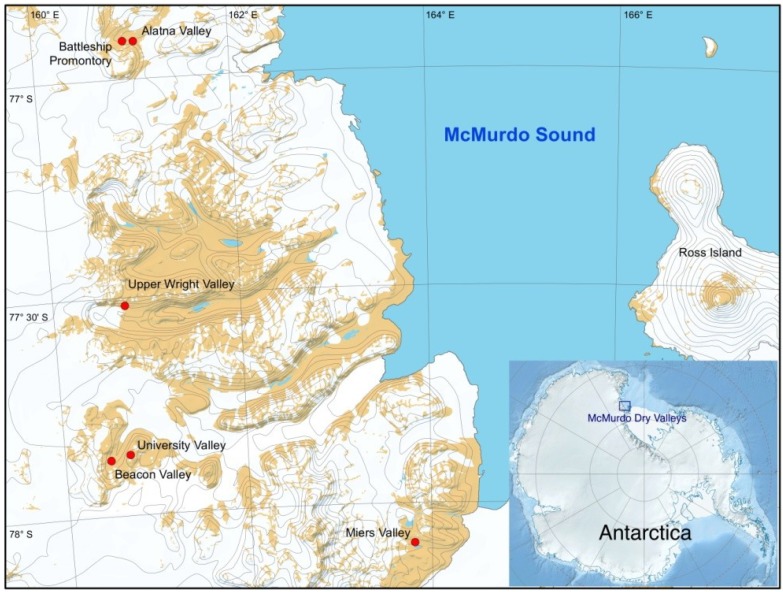
Antarctica is presented in the lower right corner, with the McMurdo Dry Valleys marked in a blue rectangle. The locations of the sampling sites within the McMurdo Dry Valleys are displayed by red dots.

Soil was collected at six different sites in the McMurdo Dry Valleys (Table 1 and Figure 1) as described previously [15]. Briefly, sampling sites were all located on a south facing, 0–20° slope. An intersection was made by two 50 m transects, with the intersection in the middle being the central sampling point (X or C). Four sampling points around the central point were marked (A–D with A being the southernmost point and the remaining points in an anti-clockwise order, or N, E, S, W). Five scoops of the top 2 cm of soil were collected and homogenized at each identified (1 m^2^) sampling point after pavement pebbles were removed. Samples were stored in sterile Whirl-Pak (Nasco International, Fort Atkinson, WI, USA) at −20 °C until returned to New Zealand, where they were stored at −80 °C until analysis.

**Table 1:** List of sampling sites.

Valley	Coordinates	Elevation	Sampling Date
Miers Valley	78°05.486'S, 163°48.539'E	171 m	December 2006
Beacon Valley	77°52.321'S, 160°29.725'E	1376 m	December 2006
Upper Wright Valley	77°31.122'S, 160°45.813'E	947 m	January 2008
Battleship Promontory	76°54.694'S, 160°55.676'E	1028 m	January 2008
Alatna Valley	76°54.816'S, 161°02.213'E	1057 m	November 2010
University Valley	77°51.668'S, 160°42.736'E	1680 m	November 2010

### 2.2. Soil Chemistry

Soil moisture content was determined by drying 6 g of soil at 35 °C until its weight stabilized and then at 105 °C until the sample reached constant weight. Soil pH and electrical conductivity were determined using the slurry technique, which is based on a 2:5 unground dried soil:de-ionized water mixture rehydrated overnight before measurement, using a Thermo Scientific Orion 4 STAR pH/Conductivity meter (Thermo Scientific, Beverly, MA, USA). For total and organic carbon and nitrogen contents, dried soils were ground to fine powders using an agate mortar and pestle and precisely weighed out to 100 mg. Samples were analyzed with an Elementar Isoprime 100 analyzer (Elementar Analysensysteme, Hanau, Germany). Sample preparation for elemental analysis was adapted from US EPA Analytical Methods 200.2 (Revision 2.8, 1994) and Lee *et al.* [15], in which ground dried soil samples were acid digested and analyzed using an E2 Instruments Inductively Coupled Plasma Mass Spectrometer (ICP-MS) (Perkin-Elmer, Shelton, CT, USA) at the Waikato Mass Spectrometry Facility following manufacturer protocols [15]. For soil grain size, 0.3–0.4 g of 2-mm-sieved dried soil was incubated overnight with 10% hydrogen peroxide. A second excess of hydrogen peroxide was then added to the sample and heated on a hotplate. Finally, 10 mL of 10% Calgon was added to the sample and left overnight before being placed in an ultrasonic bath for 5 min. Measurements were taken on a Mastersizer 2000 (Malvern, Taren Point, NSW, Australia).

### 2.3. DNA Extraction

DNA was extracted from soils using a modified version of a previously published cetyl trimethylammonium bromide (CTAB) bead beating protocol designed for maximum recovery of DNA from low biomass soils [15,28] (Supplementary Material Text). DNA quantification was done using the QuBit-IT dsDNA HS Assay Kit (Invitrogen, Carlsbad, CA, USA).

### 2.4. Terminal Restriction Fragment Length Polymorphism Analysis

Terminal restriction fragment length polymorphism analysis (tRFLP) was utilized to identify fungal community structure and relative diversity by amplifying the intergenic spacer (ITS) between the 18S and the 28S genes of the fungal *rrn* operon. PCR was performed in triplicate and pooled together to reduce stochastic inter-reaction variability. PCR master mix included 1x PCR buffer (with 1.5 mM Mg^2+^) (Invitrogen, Carlsbad, CA, USA), 0.2 mM dNTPs (Roche Applied Science, Branford, CT, USA), 0.02 U Platinum Taq (Invitrogen, Carlsbad, CA, USA), 0.25 µM of both forward and reverse primer (Custom Science, Auckland, New Zealand) (ITS1-F and 3126R; Table S1), and 0.02 mg/mL bovine serum albumin (Sigma Aldrich, St. Louis, MO, USA) and was treated with ethidium monoazide at a final concentration of 25 pg/µL to inhibit contaminating DNA in the reagents [29]. PCR was carried out using the following thermal cycling conditions: 94 °C for 3 min; 35 cycles of 94 °C for 20 s, 52 °C for 20 s, 72 °C for 1 min 15 s; and 72 °C for 5 min on a DNA Engine thermal cycler (Bio-Rad Laboratories, Hercules, CA, USA). Successful PCR was confirmed with 1% Tris-acetate-EDTA (TAE) agarose gels, and PCR products were cleaned using the Ultraclean 15 DNA Purification kit (MOBIO Laboratories, Carlsbad, CA, USA) according to manufacturer instructions. DNA was quantified using the QuBit-IT dsDNA HS Assay Kit. 40 ng of DNA was digested with 2 U of MspI and 1× restriction enzyme buffer (Roche Applied Science, Branford, CT, USA) according to manufacturer instructions and purified with Ultraclean 15 DNA Purification kit. Lengths of fluorescent-labeled PCR amplicons (*i.e.*, tRFLP fragments) were determined by capillary electrophoresis at the Waikato DNA Sequencing Facility using an ABI 3130 Genetic Analyzer (Life Technologies, Carlsbad, CA, USA) at 10 kV, a separation temperature of 44 °C for 2 h, and the GeneScan 1200 LIZ dye Size Standard (Life Technologies, Carlsbad, CA, USA).

### 2.5. 454 Pyrosequencing

PCR protocol for preparing amplicons for pyrosequencing was identical to that for tRFLP, except a different reverse primer (ITS4, Table S1) was used. PCR products were purified using gel extraction and the QuickClean 5M PCR Purification Kit (GenScript, Piscataway, NJ, USA). A second round of PCR using fusion primers containing adapters for 454 pyrosequencing was performed (Table S1). These products were purified using Agencourt AMPure XP Beads (Beckman Coulter, Inc., Brea, CA, USA) for PCR amplicon recovery and removal of unincorporated dNTPs, primers, primer dimmers, salts and other contaminants (Beckman Coulter, Beverly, MA, USA) according to manufacturer instructions. Quality of PCR amplicon libraries was checked using the Agilent High Sensitivity DNA Kit with a BioAnalyzer (Agilent 2100, Agilent Technologies, Santa Clara, CA, USA) and the Kapa Library Quantification Kit—454 Titanium (Kapa Biosystems, Wilmington, MA, USA). 454 pyrosequensing was performed using a Roche 454 Junior sequencer at the Waikato DNA Sequencing Facility following manufacturer protocols.

### 2.6. Data Analysis

Environmental variables were log(x + c) transformed, where c is the 1st percentile value for the variable (except [Ag] where c is the mean due to low values), prior to analysis; pH values were not transformed. A Euclidean distance matrix was calculated in PRIMER 6 (PRIMER-E Ltd., Ivybridge, UK) from the transformed environmental variables and used for downstream analyses. tRFLP traces were first processed using PeakScanner 1.0 (Life Technologies, Carlsbad, CA, USA) to export all peaks above 5 relative fluorescence units (RFU). The resulting profiles were further processed using an in-house collection of python and R scripts (available from authors upon request) to identify true signal peaks as well as binning peaks based on their sizes. Briefly, peaks outside the size range of 50–1200 bp were excluded from analysis, and only peaks whose heights are greater than the 99% confidence threshold (*i.e.*, alpha value of 0.01) within a log-normal distribution were considered to be non-noise. Additionally, peaks had to be greater than 50 RFU to be considered non-noise, and all peaks above 200 RFU were by default designated as non-noise peaks. Peaks were then binned to the nearest 1 bp, and only peaks whose relative abundance was greater than 0.1% were retained. The resulting matrix of peaks expressed as relative abundances was imported into PRIMER 6, and a Bray-Curtis similarity matrix was calculated for downstream analyses. Using these distance matrices, PRIMER 6 was used to generate non-metric multidimensional scaling (MDS) plots, perform group-average hierarchical clustering, and carry out one-way analysis of similarities (ANOSIM) and biota-environmental stepwise (BEST) analyses.

454 pyrosquencing flowgrams were denoised using AmpliconNoise v1.24 [30], including a SeqNoise step to remove PCR errors and a Perseus step to remove PCR chimeras [30]. Denoised reads were aligned pair-wise using ESPIRIT [31], which directly generated a distance matrix. Mothur 1.26 was used to cluster the sequences at 0.15 distance with nearest neighbor clustering [32], and the representative sequences for the resulting operational taxonomic units (OTUs) were checked (blastn with word size of 7) against the GenBank *nr* database to allow manual identification of fungal ITS sequences (>250 bp and >80% similarity to known fungal ITS sequences). The curated sequences were then re-clustered using average neighbor at 0.05 distance. OTUs with fewer than 9 reads were excluded from downstream analysis as an aggressive filter against spurious OTUs that arose from non-specific PCR amplification and sequencing errors.

## 3. Results and Discussion

### 3.1. Soil Geochemistry

**Figure 2:**
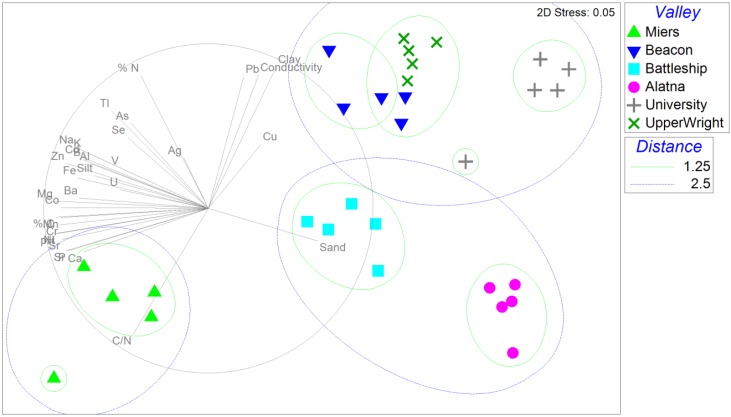
Nonmetric multidimensional scaling (MDS) plot based on Euclidean distances between soil physicochemical profiles. Significant correlations (Pearson *R* > 0.25) between plot ordinations and soil physicochemical properties are represented as vectors in gray.

Soils from six Dry Valleys were characterized as loamy sand or sand due to their low clay (<2%) and silt (<13%) contents (Table S2), which is congruent with Antarctica’s known slow and primarily physical weathering processes [7]. The coarse soil texture likely resulted from low erosivity of cold-based glaciers and salt weathering, which causes comminution of coarse fragments and provides a steady supply of sandy grains to the soils [7]. Consequently, these soils lack significant aggregation and have poor moisture retention capacity, which is consistent with their low gravimetric water content (Table S2). Water availability has been suggested to be a major factor controlling biomass and diversity of Antarctic vegetation [33,34]. Among the six study sites, Miers Valley soils contained the lowest average moisture content (0.53%, ANOVA *p*-value = 0.002; Table S2). But due to its low elevation (elev. 171 m) and variable wind direction, temperatures in Miers Valley can reach above 0 °C in austral summers [35]. This likely leads to increased water availability from melt streams of Miers and Adams Glaciers, which can trigger rapid responses from local microorganisms [16,34]. Water availability in austral summers is also elevated in Alatna Valley and Battleship Promontory, where transient ponds are formed from snow melt. This is in contrast with the low moisture content and water availability in higher (elev. >1500 m) and more inland valleys (e.g., University Valley). The high altitude of University Valley results in colder air temperatures all year round, leading to a lower net ice loss rate when compared to Beacon Valley (*ca*. 450 m below University Valley) [36]. Soil salt content is a proxy for water availability [37], and Miers Valley, Alatna Valley, and Battleship Promontory soils showed relatively low conductivity. Soil physicochemical properties (Table S2) were significantly different among the sampling sites (ANOSIM global *R* = 0.963, *p*-value = 0.001) with each valley clearly forming its own clade. In a broader view, distinct grouping patterns emerged for Miers Valley in the MDS plot (Figure 2), possibly due to its alkaline pH reflective of greater influence from salts of marine origin [38] and its higher C/N ratio. Overall, geochemical analysis revealed a wide range of soil salinity (107–3920 µS), low moisture content (1%–3% w/v), low levels of organic carbon (<0.46%) and nitrogen (<0.12%).

### 3.2. Community Fingerprinting with tRFLP

DNA extractions from soils proved difficult, and DNA samples from Beacon, University, and Upper Wright Valleys were mostly below the detection limit of 0.05 ng/µL. The highest recovery yields were obtained from Miers Valley samples, followed by those from Battleship Promontory and Alatna Valley (Table 2). Fungal tRFLP analysis of extracted DNA returned positive signals for 12 of the 30 soil samples, with no polymorphic fragments (PFs) detected in any of the samples from University Valley. A total of 33 PFs were obtained (Table 3), whose lengths varied between 145 and 781 bp. Samples from Battleship Promontory collectively returned the highest diversity (13 PFs), followed by Alatna Valley (11 PFs) and Miers Valley (5 PFs). ANOSIM analysis of PF profiles demonstrated statistically significant differences among valleys (ANOSIM global *R* = 0.731, *p*-value = 0.001), and there was no robust correlation between diversity (PF count) and biomass (averaged DNA yield from 1 gram of soil) (*R* = 0.35, *p*-value = 0.06).

**Table 2:** Average concentrations of DNA extracted from 1 g of soil.

Valley	Average Concentration ± S.D.
Miers Valley	48.60 ± 27.79 ng/µL
Beacon Valley	0.48 ± 0.55 ng/µL
Battleship Promontory	20.87 ± 5.61 ng/µL
Upper Wright Valley	3.68 ± 7.57 ng/µL
Alatna Valley	15.84 ± 13.49 ng/µL
University Valley	0.05 ± 0.09 ng/µL

**Table 3:** Summary of terminal restriction fragment length polymorphism (tRFLP) polymorphic fragments (PF).

Valley	Total PF	Average PF ± S.D.
Miers Valley	5	1.0 ± 1.2
Beacon Valley	2	0.4 *
Battleship Valley	13	2.6 ± 1.5
Wright Valley	2	0.4 *
Alatna Valley	11	2.2 ± 3.2
University Valley	0	0

* S.D. not calculated.

**Figure 3:**
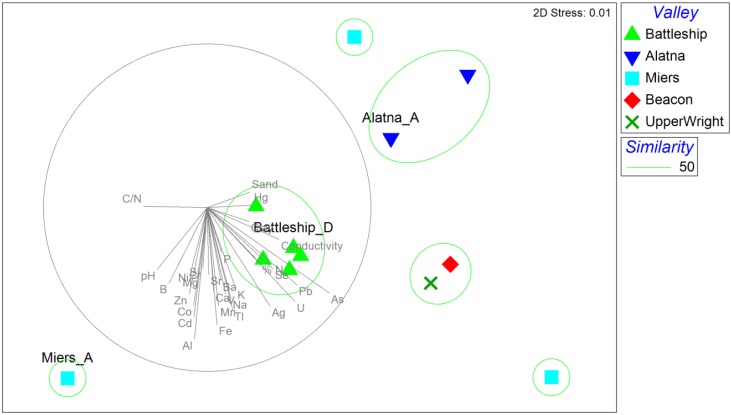
Nonmetric multidimensional scaling (MDS) plot based on Bray-Curtis similarities of tRFLP profiles. Samples used for 454 PCR amplicon pyrosequencing are labeled by name. Significant correlations (Pearson *R* > 0.25) between plot ordinations and soil physicochemical properties are represented as vectors in gray.

Interestingly, a MDS plot of tRFLP data showed a clear separation of samples from Battleship Promontory and Alatna Valley (Figure 3), despite the fact that the two sampling sites are less than 5 km apart and within line-of-sight. This suggests that aeolian dispersal between these sites is very limited or outweighed by other environmental drivers that shape edaphic fungal diversity at these locations. There was only one sample each from Beacon and Upper Wright Valleys, but they were >50% similar to each other. Samples from Miers Valley were widely dispersed in the MDS plot, making Miers Valley a clear outlier.

### 3.3. 454 Pyrosequencing

**Figure 4:**
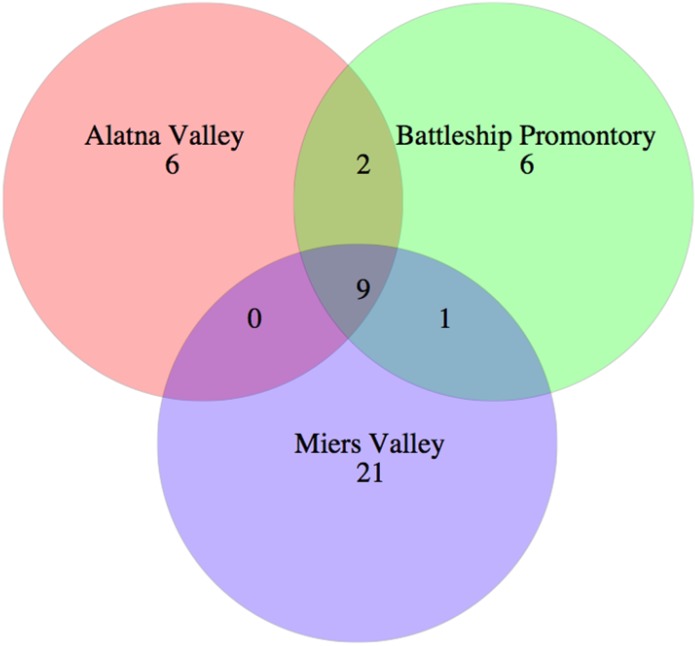
Venn diagram of fungal OTUs.

To identify the fungal species present, three samples that represented the greatest diversity based on results from tRFLP analysis were chosen for 454 PCR amplicon pyrosequencing. DNA extracted from Battleship Promontory sample D, referred to as Battleship_D, Alatna Valley sample N (Alatna_N), and Miers Valley sample A (Miers_A) appeared to be most representative of each major cluster (Figure 3). Fungal signals in Beacon and Upper Wright Valley were considered unsequenceable due to very low DNA extraction and amplification yields and therefore excluded from pyrosequencing. After filtering, denoising, chimera removal, and quality control, 262 fungal OTUs (from 21,101 reads) were obtained, of which 37 contained more than 9 reads (*i.e.*, >0.2% of the sample with fewest reads) and were used for downstream analysis. Species richness (Table 4) was highest in Miers Valley (31 OTUs from 1771 reads), followed by Battleship Promontory (18 OTUs from 2091 reads), and Alatna Valley (17 OTUs from 5081 reads). A Venn diagram illustrates the distribution of OTUs among the three samples (Figure 4). Nine OTUs (representing 8943 reads) were found in all three Valleys (Figure 4), including the five most abundant OTUs.

**Figure 5:**
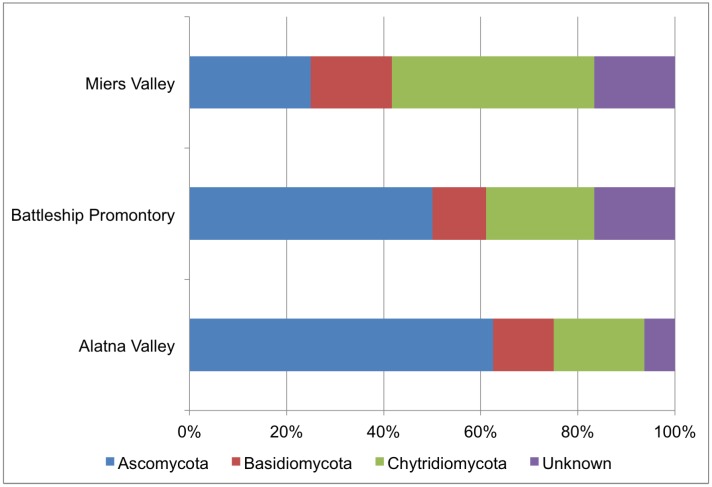
Phylum-level distribution of fungal OTUs.

A significant number of OTUs were annotated as unclassified (Table 4 and Figure 5), which is likely reflective of the comparative lack of high quality annotated fungal ITS sequences in the GenBank *nr* database. Therefore, results that rely on classification of fungal sequences must be interpreted carefully. However, multiple studies identified Ascomycota and Basidiomycota as the dominant fungal phyla in the Dry Valleys [22,25,27,39], whereas our results showed an unexpected prominence of Chytridiomycota among all three valleys (Figure 5). It should be noted that Chytridiomycota were reported in a molecular survey on west Antarctic sites [40], including Signy Island, Mars Oasis, and Coal Nunatak, at significant abundances but not in the Dry Valleys.

**Table 4:** Overview of fungal OTUs from PCR amplicon pyrosequencing.

	Read Count				Best Match in GenBank *nr* Database			
OTU #	AV_N	BP_D	MV_A	Total	GenBank ID	Identity (%)	Phylum	Organism
3	1852	407	1	2283	AB032673	99	Basidiomycota	*Cryptococcus consortionis*
4	841	728	191	1760	EF432821	93	Chytridiomycota	*Lobulomycetales* sp. AF017
6	1058	122	369	1542	EF060799	99	Ascomycota	*Herpotrichiellaceae* sp. LM500
7	505	68	233	806	JF747078	99	Ascomycota	*Exophiala equina*
10	129	351	61	541	EU480339	93	Unknown	Uncultured clone
11	0	0	372	372	GQ250013	92	Ascomycota	*Cordyceps* sp. BCC22921
14	246	0	0	246	EF535204	90	Ascomycota	*Candelaria crawfordii* strain CHN265
16	179	0	0	179	FJ827708	90	Chytridiomycota	*Powellomyces* sp. PL 142
20	0	109	0	109	EU352772	93	Chytridiomycota	*Chytridiales* sp. JEL178
22	0	0	109	109	DQ457086	85	Unknown	Uncultured clone
24	0	83	0	83	AM901700	97	Ascomycota	*Ascomycete* sp. BF104
25	0	0	81	81	FJ827708	94	Chytridiomycota	*Powellomyces* sp. PL 142
26	80	0	0	80	GU184116	96	Ascomycota	*Acarospora rosulata* isolate ACABUL_USA2
28	36	30	0	66	KC222134	83	Ascomycota	*Trichoglossum octopartitum*
29	0	0	61	61	EF585664	83	Chytridiomycota	*Betamyces americaemeridionalis*
35	0	0	54	54	EU352770	92	Chytridiomycota	*Lobulomyces poculatus*
39	0	47	0	47	AF106527	91	Ascomycota	*Arthrobotrys arcuata* strain CBS 174.89
40	8	33	1	42	DQ494379	94	Ascomycota	*Vermispora fusarina*
41	12	27	3	42	JX171180	94	Basidiomycota	*Meira* sp. ANTCW08-165
45	34	1	5	40	FJ827741	96	Chytridiomycota	*Gaertneriomyces* sp. JEL 550
48	29	1	0	30	HQ634632	97	Ascomycota	*Chaetothyriales* sp. M-Cre1-2
49	29	0	0	29	JX124723	98	Ascomycota	*Taphrina* sp. CCFEE 5198
51	0	0	28	28	JX036093	93	Ascomycota	*Polysporina frigida*
54	0	10	17	27	EU352770	92	Chytridiomycota	*Lobulomyces poculatus*
56	0	0	25	25	JF809853	99	Chytridiomycota	*Betamyces* sp. PL 173
59	0	0	23	23	AY373015	91	Unknown	*Olpidium brassicae*
60	0	22	0	22	JQ936330	99	Unknown	*Phaeosphaeriopsis* sp. CBP21E
61	0	0	22	22	JX219783	91	Ascomycota	*Cortinarius callisteus*
62	0	0	22	22	JN416510	89	Basidiomycota	*Basidiobolus* sp. BCU1
64	1	19	1	21	JX173100	99	Ascomycota	*Cladosporium* sp. AF13
67	18	0	0	18	AY781244	89	Unknown	*Ascomycete* sp. olrim401
68	0	18	0	18	AY394892	94	Ascomycota	*Mycorrhizal* sp. pkc11
72	0	0	17	17	EF634250	80	Chytridiomycota	*Coralloidiomyces digitatus*
78	0	15	0	15	EU480016	90	Unknown	Uncultured clone
101	0	0	11	11	JN882333	94	Chytridiomycota	*Monoblepharis hypogyna*
102	0	0	11	11	DQ485612	93	Chytridiomycota	*Rhizophydium carpophilum*
105	0	0	10	10	JQ711836	99	Basidiomycota	*Russula nigricans*

Abbreviations: OTU, operational taxonomic unit; AV_N, Alatna Valley sample N; BP_D, Battleship Promontory sample D; MV_A, Miers Valley sample A.

Contrary to fungal tRFLP results, PCR amplicon pyrosequencing analysis of the fungal ITS region identified Miers Valley as having the highest level of diversity of the three valleys (Figure 4), despite the lowest sequencing depth. In particular, Miers Valley appeared to harbor a limited presence of Ascomycota compared to the other two valleys, but also the highest number of Chytridiomycota OTUs (Figure 5).

The most abundant OTU (#3) was found in Alatna Valley (1875 reads), Battleship Promontory (407 reads), and Miers Valley (1 read) (Table 4). Its best match in GenBank (99% identity) was the psychrotolerant species *Cryptococcus consortionis* (Basidiomycota), which was previously observed and commonly found in Dry Valley soils [22,41]. *Cryptococcus consortionis* is characterized by the combination of amylase production and inability to utilize nitrate, cellobiose, D-galactose, *myo*-inositol, and mannitol [41]. The second most abundant OTU (#4) was also found in all three Dry Valleys (Table 4). Its best match in GenBank (93% similarity) was *Lobulomycetales* sp. AF017 (Chytridiomycota), which has been reported to occur in barren alpine soil in Peru [42]. Two other OTUs (#35 and #54) appeared to be affiliated with this genus as well.

Other abundant OTUs found in all three valleys (Table 4) were 99% similar to the species *Herpotrichiellaceae* sp. LM500 (Ascomycota) and 99.9% similar to *Exophiala equine* (Ascomycota), which was curiously reported to occur exclusively in waterborne cold-blooded animals [43]. Less abundant OTUs show similarity to fungal taxa described as Dry Valley lichen *Polysporina frigida* [44], *Meira* sp. ANTCW08-165 [45], and *Tetracladium* sp. ANTCW08-156 [45] which were previously detected in Antarctica. The genus *Cladosporium* has been reported as a dominant group by multiple studies [24,46,47] of pristine areas with little biotic influence [24,46], likely because of its prolific production of spores and high abundance in the air [24,47]. This is in contrast to our study, where *Cladosporium* species appear to be very rare (21 reads total). Notably, these fungi have been reported to survive repeated inoculations [24] and form spores, which can remain dormant for considerable periods of time [26]. It should be stressed that no conclusions can be drawn as to whether these fungi are active based on PCR amplicon pyrosequencing, as the method only detects the presence of DNA and does not indicate the viability of the organism [48,49].

### 3.4. Biogeography and Local Adaptation

The most important dispersal mechanisms for biomass in Antarctica have been suggested as aeolian transport [4,50,51]. If, as hypothesized previously [52], fungal species in the Dry Valleys are inactive spores that only respond to cultivation efforts and do not exhibit localized adaptations, neighboring valleys would be expected to harbor very similar fungal communities; for example, between Battleship Promontory and Alatna Valley and between Beacon and University Valley, which are located next to each other (<1 km) without any physical barrier. The tRFLP results indicated highly localized community structures, with Battleship Promontory and Alatna Valley forming statistically distinct clades (Figure 3). In addition, no fungal signals were detected in samples from University Valley while some were detected in Beacon Valley samples. Rao *et al.* previously hypothesized that the biogeography may be important for fungi in the Dry Valleys [52] and that fungal tolerance to saline conditions could confer selective advantage in high-elevation Dry Valleys [52]. Although the five most abundant OTUs reported here were found in all three samples sequenced, the relative abundances of individual OTUs were highly divergent. Since each of the sequenced samples can be considered representative of distinct diversity patterns found in the three Dry Valleys (Figure 3), the relative abundance patterns suggest that distinct fungal communities exist in each of these locations (Table 4). It should be noted that the limited spatial coverage in each Dry Valley and lack of replicates for sequencing analysis preclude definitive conclusions from being drawn, but these observations could indicate that aeolian transport plays a less important role than previous believed, or that Dry Valley fungal communities exhibit adaption to local conditions and thus are ecologically relevant.

### 3.5. Environmental Drivers of Fungal Distribution

Whether and how environmental factors shape fungal communities in Dry Valleys soils remains largely unexplored, but it has been suggested that both contemporary environmental conditions and historical contingencies play important roles in the distribution of fungal taxa in general [53]. It has been shown that abiotic factors play the most dominant role in extremely simplified food webs [5,11,54,55]. This makes the Dry Valleys soil ecosystem, with its extreme environmental stress, an excellent model for resolving the influence of abiotic factors on soil microbiota [19,55,56]. Miers Valley and Battleship Promontory, whose soils generally have a lower salinity, were reported to harbor greater bacterial and cyanobacterial diversity [15]. This study reveals similar trends for edaphic fungal diversity in these Dry Valleys as well as Alatna Valley; compared with Beacon Valley, University Valley, and Upper Wright Valley, where the lack of amplifiable fungal signal in extracted DNA could indicate potential limits of fungal growth and distribution. Importantly, soil C/N ratios are higher in all three coastal and lower elevation valleys, which potentially indicate higher levels of primary productivity that can in turn sustain diverse populations of heterotrophic fungi [4,16,57]. Rao *et al.* suggested that substrate availability could limit diversity [52], since Dry Valley soils with higher carbon content harbored greater species richness [22,52]. Biota-environmental stepwise (BEST) analysis of soil physicochemical properties and tRFLP results supported this view, identifying C/N ratio as the most consistent differentiator of fungal community structure, followed by As and Ca (Supplementary Table S3). Calcium can be considered as a proxy for the mineral composition of underlying soils. The influence of arsenic on fungal populations is not clear since its concentrations are very low in our samples (Supplementary Table S2). The complete/near absence of detectable fungal signal in samples from University Valley and Beacon Valley is intriguing. Compared with other valleys, Beacon Valley and University Valley have higher elevations, resulting in lower average temperature and possibly less ice melting [36]. Therefore, contrary to an earlier hypothesis [52], lower temperature and water availability, combined with lower C/N ratio and higher salinity, may create conditions in these inland Dry Valleys that restrict fungal growth while permitting bacterial presence [15]. However, given that our samples were taken within comparatively small areas (2500 m^2^) on south-facing slopes, the possibility that our observations are reflective of specific geographic features of the sampling sites cannot be ruled out. South-facing slopes of the Dry Valleys are generally colder due to the lack of solar radiation input [1] and possibly more oligotrophic (compared with north-facing slopes) [16], and as such may restrict the colonization and growth of fungi.

## 4. Conclusions

Soil physicochemical properties among the Dry Valley sites showed distinct grouping patterns, with each valley forming its own clade. tRFLP results revealed similar grouping patterns, with significant variations in relative abundances of fungal signals between sites. Miers Valley was identified as a clear outlier by geochemical and tRFLP analyses, which were corroborated by pyrosequencing results, showing that Miers Valley harbored the highest level of fungal diversity and an unexpected abundance of Chytridiomycota. This is in contrast with the relatively low abundance of Basidiomycota, which was previously reported as the most dominant fungal phyla in the Dry Valleys. In total, nine OTUs were found in all three valleys, including the five most abundant ones, indicating that a set of core fungal species is present throughout the Dry Valleys. However, the relative abundances of these dominant OTUs are notably different among the three sites, suggesting that there is significant biogeography for Dry Valley edaphic fungi and that they likely respond and adapt to local environmental conditions. This in turn implies that much of the fungal biomass in the Dry Valleys is biological active and ecologically relevant, rather than spores whose distribution pattern is largely dictated by aeolian transport. The comparative lack of fungal signals in the inland high elevation Dry Valleys suggests that environmental conditions at those locations may represent limits of fungal growth.

## Supplementary Files

Supplementary File 1
