# Supplementary material for: The Distribution and Identity of Edaphic Fungi in the McMurdo Dry Valleys
Source: Biology (Basel). 2014 Jul 30;3(3):466–83. doi: 10.3390/biology3030466 (PMC4192622; doi:10.3390/biology3030466)
Supplement: Supplementary File 1 [file biology-03-00466-s001.pdf]

Supplementary Materials

DNA Extraction

Soils (0.6–1.0 g) were aseptically measured into 1.5 mL screw-cap polypropylene tubes containing 0.5 g each of 0.1 and 2.5 mm zirconia/silica beats (BioSpec Products Inc, Bartlesville, OK, USA). Beads were baked at 250 °C for 4 h prior to use. 300 µL phosphate buffer (100 mM NaH_2PO_4) and 300 µL Sodium Dodecyl Sulfate (SDS) lysis buffer (100 mM NaCl, 500 mM Tris pH 8.0, and 10% SDS) were added. This was shaken at 4.2 ms^{-1} for 30 s on a MiniBeadBeater (Glen Mills Inc, Clifton, NJ, USA), mixed on a Vortex Genie (MO BIO Laboratories, Inc. Carlsbad, CA, USA) for 10 min and centrifuged at 16100 RCF for 3 min. The supernatant was transferred to a new tube containing 200 µL hexadecyltrimethylammonium bromide (CTAB) buffer (100 mM Tris-HCl pH 8.0, 1.4 M NaCl, 25 mM EDTA, 2% CTAB, 1% polyvinylpryrrolidone, 0.04% v/v β-mercapto-ethanol) and incubated at 300 rpm 60 °C for 30 min in a Thermomixer Comfort (Eppendorf, Hamburg, Germany). The upper aqueous layer was removed to a new 1.5 mL sterile Eppendorf tube and a equal volume of chloroform: isomyl alcohol was added, vortexed for 10 s and left on a rocking bed for 20 min at room temperature. Afterwards it was pelleted by 5 min centrifugation at 16100 RCF. The aqueous fraction was removed to a new tube. DNA was precipitated with 7 M ammonium acetate to a final concentration of 2.5 M, mixed by repeated inversion and centrifuged for 5 min at 16100 RCF. The upper aqueous layer was transferred to a new sterile 1.7 mL Eppendorf tube and 0.54 volume of isopropanol was added, mixed by repeated inversion, and incubated at −20 °C overnight. The tubes were centrifuged at 16100 RCF for 20 min, the supernatant was discarded and the pellet was washed with 1 mL of ice-cold ethanol (−20 °C) and centrifuged at 16100 RCF for 30 s. The supernatant was discarded and the pellet was dried in a SpeedVac concentrator (BioLab, Dublin, OH, USA) on manual and a medium temperature. DNA was resuspended in 20 µL sterile LO-TE (3 mM Tris-HCl pH 8, 0.2 mM EDTA), and its concentration quantified using the Qubit-IT ds-DNA HS Assay Kit (Invitrogen, Carlsbad, CA, USA) and stored at −80 °C till use. Procedural blanks were included at appropriate steps and yielded no DNA detectable by PCR.

Table S1. An overview of primers used for tRFLP and 454 pyrosequencing. For sequencing, 2 sets of primers were used, ITS1-F (forward primer) and ITS4 (reverse primer) were used in the first round of PCR, and fusion primer versions of these primers (*i.e.*, with 454 sequencing adapter and barcode added) in the second round.

Name	Sequence	Analysis
ITS1-F	5'-FEM-CTTGGTCATTTAGAGGAAGTAA-3'	tRFLP
3126R	5'-ATATGCTTAAGTTCAGCGGGT-3'	tRFLP
ITS1-F	5'-CTTGGTCATTTAGAGGAAGTAA-3'	454 Pyrosequencing—1st PCR
ITS4	5'-TCCTCCGCTTATTGATATGC-3'	454 Pyrosequencing—1st PCR
UoW1_ITS1F	5'-CCATCTCATCCCTGCGTGTCTCCGACTCA GCGAGTCTTGGTCATTTAGAGAAGTAA-3'	454 Pyrosequencing—2nd PCR
UoW3_ITS1F	5'-ATCTCATCCCTGCGTGTCTCCGACTCAGA CGATACGCTTGGTCATTTAGAGGAAGTAA-3'	454 Pyrosequencing—2nd PCR
UoW4_ITS1F	5'-CATCTCATCCCTGCGTGTCTCCGACTCA GCTGACCTTGGTCATTTAGAGGAAGTAA-3'	454 Pyrosequencing—2nd PCR
UoW12_ITS1F	5'-ATCTCATCCCTGCGTGTCTCCGACTCAGC AGTACGCTTGGTCATTTAGAGGAAGTAA-3'	454 Pyrosequencing—2nd PCR
UoW_ITS4	5'-CTATCCCCTGTGTGCCTTGGCAGTCTCAGTC CTCCGCTTAT-3'	454 Pyrosequencing—2nd PCR

Table S2. Soil geochemical properties (S.D. in parentheses).

	**Miers Valley**	**Beacon Valley**	**Battleship Promontory**	**Upper Wright Valley**	**Alatna Valley**	**University Valley**
Clay: 0.06–2 µm (%)	0.02 (0.01)	2.42 (1.05)	0.82 (0.72)	6.43 (2.83)	0.13 (0.09)	2.18 (0.85)
Silt: 2–63 µm (%)	28.38 (7.04)	13.52 (5.29)	8.22 (4.92)	14.82 (8.83)	5.39 (1.38)	9.96 (2.63)
Sand: 63–2000 µm (%)	71.61 (7.04)	84.06 (6.24)	90.6 (5.62)	78.69 (7.37)	94.48 (1.42)	87.86 (3.10)
pH	8.62 (0.31)	7.1 (0.28)	7.68 (0.51)	6.96 (0.09)	6.31 (0.06)	6.64 (0.47)
Conductivity (µS)	300	3920	107	6130	194.46	26917
Gravimetric Water Content (%)	0.53 (0)	2.36 (0.16)	1.13 (0.05)	1.07 (0.03)	1.16 (0.01)	0.67 (0)
C%	0.46 (0.02)	0.14 (0.04)	0.1 (0.00)	0.11(0.10)	0.05 (0.01)	0.04 (0.00)
N%	0.05 (0.02)	0.08 (0.01)	0.04 (0.01)	0.12 (0.02)	0.01 (0.01)	0.03 (0.00)
C/N	18.22 (20.07)	1.8 (0.46)	2.5 (0.25)	0.98 (0.23)	6.12 (1.08)	1.31 (0.44)
Ag	0.03 (0.04)	0.04 (0.06)	0.00 (0)	0.00 (0.00)	0.00 (0.00)	0.05 (0.09)
Al	23441 (4853)	27273 (7267)	24690 (3110)	20033 (4926)	11920 (1599)	12731 (1971)
As	1.86 (1.43)	2.2 (0.94)	1.17 (0.28)	1.95 (0.22)	1.03 (0.30)	0.97 (0.13)
B	1011 (49)	1014 (56)	1038 (42)	1028 (32)	64 (64)	40 (5)
Ba	150 (40)	55 (40)	25 (4)	40 (8)	21 (2)	42 (10)
Ca	24673 (5.3)	16187 (4.4)	9813 (1.1)	5494 (2.0)	8392 (1.2)	7177 (1.0)
Cd	0.25 (0.05)	0.21 (0.08)	0.13 (0.04)	0.17 (0.08)	0.06 (0.02)	0.09 (0.06)
Co	30.88 (6.1)	22.34 (3.1)	16.12 (2.5)	12.53 (2.4)	9.9 (1.3)	12.09 (2.1)
Cr	51.84 (10.02)	10.97 (3.58)	7.46 (0.67)	8.61 (1.41)	4.61 (0.67)	7.55 (0.98)
Cu	23.89 (5.4)	147.33 (25.2)	99.7 (15.9)	66.16 (11.2)	63.66 (7.5)	59.9 (13.8)
Fe	44330.21 (8.5)	48771.24 (10.9)	31943.47 (3.8)	27758.07 (4.0)	21511.96 (2.6)	22195.61 (3.3)
Hg	5157.3 (1143)	2098.73 (883)	1026.13 (133)	3752.67 (2115)	695.2 (93)	1514.37 (171)
K	40689.52 (6816)	10561.11 (2142)	5524.19 (745)	7881.83 (1395)	2589.6 (291)	5457.38 (891)
Mg	733504 (136)	425.26 (97)	317.42 (46)	300.56 (91)	241.25 (39)	267.95 (35)
Mn	7767.4 (1384)	4426.65 (1454)	2799.42 (429)	7030.73 (3546)	1463.64 (201)	2186.25 (363)
Na	169.63 (31.49)	29.02 (5.62)	22.51 (3.18)	18.15 (2.57)	9.93 (1.31)	16.05 (2.53)
Ni	1750.61 (334)	974.41 (177)	719.47 (113)	517.38 (103)	580.48 (68)	404.82 (72)
P	4216 (1.26)	8846 (1.97)	3.62 (0.51)	9138 (1.72)	4.7 (0.75)	8.31 (1.02)
Pb	0.96 (0.26)	1.58 (0.38)	1.16 (0.23)	1.13 (0.15)	0.62 (0.07)	0.51 (0.08)
Se	12387.92 (3726)	1886.23 (77)	1158 (125)	1888.86 (217)	1350.81 (118)	1424.45 (75)
Si	321.11 (82.26)	70.45 (20.19)	37.21 (4.81)	32.43 (7.81)	27.03 (3.49)	29.85 (4.05)
Sr	1.17 (0.29)	1094 (0.19)	0.89 (0.12)	1.05 (0.45)	0.89 (0.10)	0.77 (0.06)
U	78.39 (15.78)	155.82 (57.07)	80.72 (9.22)	64.59 (12.02)	48.01 (9.18)	34.62 (4.15)
V	104.44 (6.99)	104.13 (22.08)	63.28 (9.50)	64.61 (9.76)	35.01 (4.49)	43.04 (4.55)
Zn	0.02 (0.01)	2.42 (1.05)	0.82 (0.72)	6.43 (2.83)	0.13 (0.09)	2.18 (0.85)

All values were calculated from five sampling points from each valley (except for gravimetric water content, where only sampling points A and C were measured for Miers, Beacon, Upper Wright Valley and Battleship Promontory). Elemental concentrations are in ppm unless otherwise noted.

Table S3. Results from BEST analysis.

Correlation Coefficient (Rho)	Variables
0.698	C/N, Al, As, Ca, Mn
0.697	C/N, Al, As, Ca, Pb
0.695	C/N, As, Ca, Pb
0.695	Sand, C/N, Al, As, Ca
0.695	C/N, Al, As, Ca
0.694	Sand, C/N, Al, As
0.694	C/N, As, Co, Mn, Pb
0.694	C/N, As, Ca, Fe, Pb
0.694	C/N, As, Ca
0.694	C/N, As, Ca, Co, Pb

© 2014 by the authors; licensee MDPI, Basel, Switzerland. This article is an open access article distributed under the terms and conditions of the Creative Commons Attribution license (http://creativecommons.org/licenses/by/3.0/).
